# New Imaging Modality of COVID-19 Pneumonia Developed on the Basis of Alzheimer’s Disease Research

**DOI:** 10.3390/ijms23158405

**Published:** 2022-07-29

**Authors:** Przemysław Koźmiński, Dorota Niedziałek, Grzegorz Wieczorek, Paweł K. Halik, Kamila Czarnecka, Aleksandra Rogut, Łukasz Cheda, Zbigniew Rogulski, Paweł Szymański, Ewa Gniazdowska

**Affiliations:** 1 Centre of Radiochemistry and Nuclear Chemistry, Institute of Nuclear Chemistry and Technology, 03-195 Warsaw, Poland; p.halik@ichtj.waw.pl (P.K.H.); e.gniazdowska@ichtj.waw.pl (E.G.); 2 Institute of Biochemistry and Biophysics, Polish Academy of Sciences, 02-106 Warsaw, Poland; dniedzialek@ibb.waw.pl (D.N.); gigo@ibb.waw.pl (G.W.); 3 Department of Pharmaceutical Chemistry, Drug Analyses and Radiopharmacy, Medical University, 1 Muszynskiego St., 90-151 Lodz, Poland; kamila.czarnecka@umed.lodz.pl (K.C.); aleksandra.rogut@stud.umed.lodz.pl (A.R.); 4 Faculty of Chemistry, University of Warsaw, Pasteura 1, 02-093 Warszawa, Poland; lcheda@chem.uw.edu.pl (Ł.C.); rogul@chem.uw.edu.pl (Z.R.); 5 Department of Radiobiology and Radiation Protection, Military Institute of Hygiene and Epidemiology, 4 Kozielska St., 01-163 Warsaw, Poland

**Keywords:** tacrine, COVID-19, gallium-68, radiopharmaceuticals, biodistribution studies, molecular docking

## Abstract

Viral pneumonia caused by highly infectious SARS-CoV-2 poses a higher risk to older people and those who have underlying health conditions, including Alzheimer’s disease. In this work we present newly designed tacrine-based radioconjugates with physicochemical and biological properties that are crucial for the potential application as diagnostic radiopharmaceuticals. A set of ten tacrine derivatives was synthesized, labelled with gallium-68 and fully characterized in the context of their physicochemical properties. Based on these results, the final two most promising radioconjugates, [^68^Ga]Ga-NODAGA-Bn-NH(CH_2_)_9_Tac and [^68^Ga]Ga-THP-NH(CH_2_)_9_Tac, were selected for biodistribution studies. The latter compound was proven to be a good inhibitor of cholinesterases with significant affinity toward the lungs, according to the biodistribution studies. On the basis of molecular modelling combined with in vitro studies, we unraveled which structural properties of the developed tacrine derivatives are crucial for high affinity toward acetylcholinesterase, whose increased levels in lung tissues in the course of coronavirus disease indicate the onset of pneumonia. The radiopharmaceutical [^68^Ga]Ga-THP-NH(CH_2_)_9_Tac was ultimately selected due to its increased accuracy and improved sensitivity in PET imaging of lung tissue with high levels of acetylcholinesterase, and it may become a novel potential diagnostic modality for the determination of lung perfusion, including in inflammation after COVID-19.

## 1. Introduction

The increasing occurrence of neurodegenerative disorders such as Alzheimer’s disease (AD) has become a significant challenge worldwide. The devastation of the cognitive functions of the AD-affected brain begins with short-term memory loss, and through slow desolation of communication capabilities, problems with orientation, moves to apathy, complete immobility and, as a consequence, loss of bodily functions and death. The most common cause of death among Alzheimer’s patients are infections, especially those causing pneumonia [1].

The coronavirus disease 2019 (COVID-19), caused by the severe acute respiratory syndrome coronavirus 2 (SARS-CoV-2) is considered one of the most infectious respiratory diseases known [2,3]. It is responsible for the worst pandemic in scale and speed of this century, associated with the highest number of global deaths, particularly among people older than 65 years, and those with underlying medical conditions, including neurodegenerative diseases [4]. Research shows that people with dementia and Alzheimer’s disease are also more likely to contract COVID-19 than people without dementia [5]. Additionally, some COVID-19 reports reveal an accelerated risk of developing Alzheimer’s disease and other dementias as a consequence of SARS-CoV-2 infections [6,7].

The method commonly used to diagnose inflammation in the course of COVID-19 is currently X-ray and computed tomography [8]. However, there are several reports of the promising application of positron emission tomography/computed tomography (PET/CT) in the detection of pneumonia associated with SARS-CoV-2 [9,10,11,12,13]. PET/CT is a non-invasive nuclear imaging technique, which allows for accurate location of the pathological tissues [14] on the basis of specific compounds (tracers) that bind to biological indicators (biomarkers).

Tacrine (1,2,3,4-tetrahydroacridin-9-amine, tetrahydroacridine, Tac) is the active substance of an oral medicament used to treat patients with Alzheimer’s disease. Tacrine belongs to the class of drugs known as cholinesterase (AChE) inhibitors and it is known for its strong and reversable binding to AChE’s catalytic site. To overcome the toxicity of tacrine, many efforts were made after its withdrawal from the market to decrease its side effects and to synthesize its new derivatives. Most of these novel tacrine-based compounds showed beneficial activities both in vitro and in vivo, and their high efficiency demonstrated that tacrine scaffold is an ideal starting point for designing and achieving potent and selective AChE’s ligands [15,16,17]. Tacrine and its analogues labelled with diagnostic radionuclide were also studied from point of view of their application as a potential diagnostic agent capable of determining AChE levels. Due to presence of AChE in the brain, liver, intestines and in glial tissues in general, tacrine and its radiolabelled analogues can also play the role of diagnostic tool to determine the physiological condition of these organs [18].

The reports on increased levels of AChE in inflamed lung tissues during coronavirus disease [19,20] motivated us to also evaluate the possibility of using of labelled tacrine derivatives to assess the physiological state of the lungs during and after the course of the coronavirus disease. Moreover, PET imaging of lungs can be used not only in diagnostics but also to assess the pulmonary disposition of drugs and the pulmonary drug transporters’ activity under physiological conditions [21,22,23].

The most commonly used radionuclide in PET/CT method is gallium-68 (^68^Ga) with a half-life ~68 min [24], which can be easily obtained from a ^68^Ge/^68^Ga generator every 4–5 h at the site of application.

The design of a PET radiotracer requires extensive basic and preclinical research to maximize the affinity and selectivity of radiolabelled molecules toward a target of the diagnosed illness biomarker (e.g., a protein overexpressed in the pathological tissues) via careful functionalization of the ligand [25,26].

In this work, we propose a new approach to labeling tacrine derivatives with ^68^Ga using various chelators. The aim of the study is to assess whether the new radiopreparations can be used both for the early diagnosis of AD disease, even before the appearance of morphological symptoms, and for a sensitive method of lung imaging modality with an early onset of viral pneumonia in the course of COVID-19. On the one hand, the design and development of a ^68^Ga-labelled PET tracers that target inflamed lung tissues appear crucial for fast implementation of proper treatment for patients at risk, for example those suffering from Alzheimer’s disease or other neurodegenerative conditions. On the other hand, a precise assessment of possible inflammation in the lungs of asymptomatic patients is necessary to assess risk of complications, including neurodegeneration.

## 2. Results

### 2.1. Syntheses of Conjugates and Radioconjugates

**Scheme 1:**
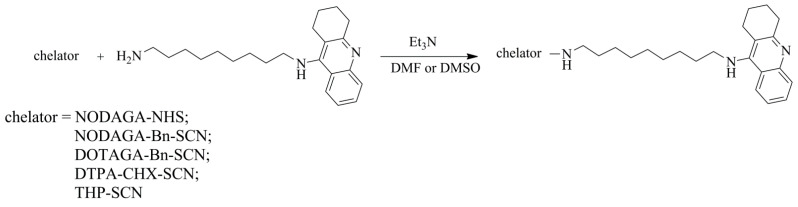
The coupling reaction of chelator with NH_2_(CH_2_)_9_Tac scaffold.

All chelator-NH(CH_2_)_9_Tac conjugates were synthesized according to Scheme 1 and the progress of reaction was tested by HPLC method. The retention times (t_R_) of the obtained conjugates are presented in Table 1. The separated and purified conjugates were confirmed by MS.

**Figure 1:**
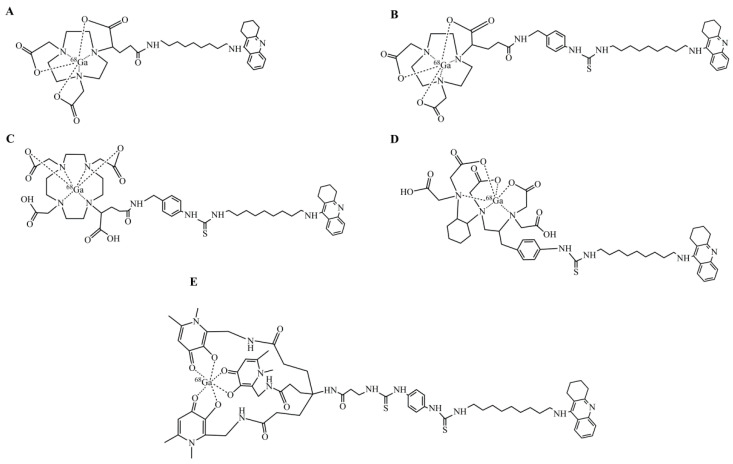
Chemical structures of the developed and investigated radioconjugates: (**A**)—[^68^Ga]Ga-NODAGA-NH(CH_2_)_9_Tac, (**B**)—[^68^Ga]Ga-NODAGA-Bn-NH(CH_2_)_9_Tac, (**C**)—[^68^Ga]Ga-DOTAGA-Bn-NH(CH_2_)_9_Tac, (**D**)—[^68^Ga]Ga-DTPA-CHX-NH(CH_2_)_9_Tac, (**E**)—[^68^Ga]Ga-THP-NH(CH_2_)_9_Tac.

**Scheme 2:**
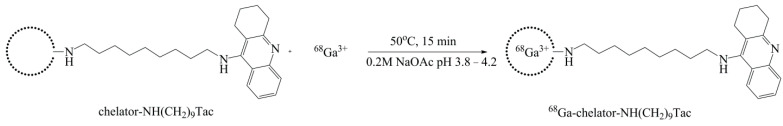
Scheme of labelling reaction of chelator-NH(CH_2_)_9_Tac conjugates.

**Figure 2:**
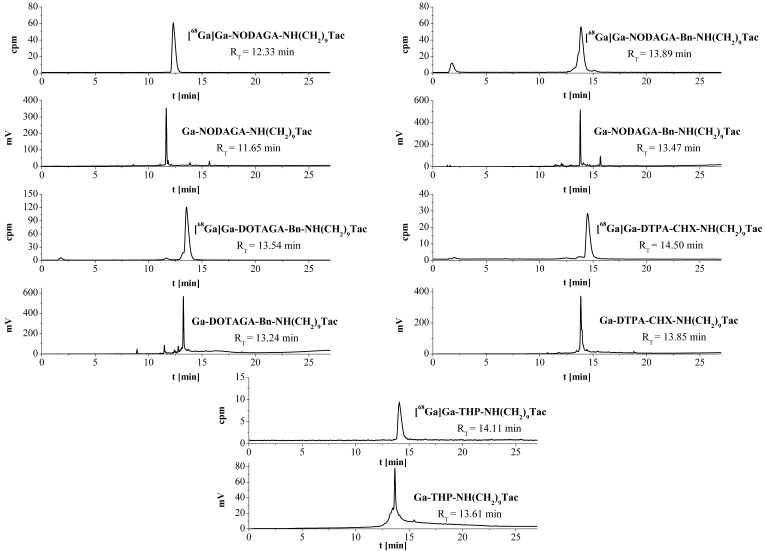
HPLC chromatograms of reaction mixture of the obtained radioconjugates and their reference compounds.

The set of five potential diagnostic tacrine-based radiopharmaceuticals (Figure 1) has been obtained according to the procedure described in Gniazdowska et al. [27]. The gallium labelling of chelator-NH(CH_2_)_9_Tac conjugates were performed according to Scheme 2. All radioconjugates have been synthesized with high yield (>90%) and purity (Figure 2). t_R_ values as well as logD_7.4_ values of all obtained radioconjugates are presented in Table 1. To verify the identity of radioconjugates, the non-radioactive reference compounds have been synthesized and characterized by HPLC method (Table 1) and mass spectrometry analysis. The HPLC chromatograms of the obtained radioconjugates, as well as reference compounds, are presented in Figure 2. Slight differences between the t_R_ values of a given radioconjugate and the corresponding reference compound result from the serial connection of UV–Vis and gamma detectors (gamma detector after the UV–Vis detector).

### 2.2. Radioconjugates Stability Studies in Human Serum

**Figure 3:**
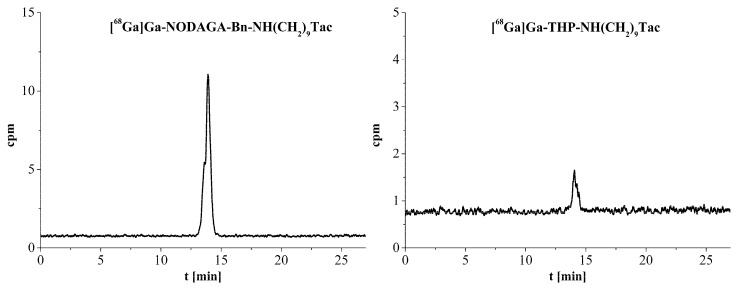
The HPLC radiochromatograms of [^68^Ga]Ga-NODAGA-Bn-NH(CH_2_)_9_Tac and [^68^Ga]Ga-THP-NH(CH_2_)_9_Tac radioconjugates recorded after 5 h of incubation at 37 °C in human serum.

For all synthesized radioconjugates, stability studies in human serum (HS) were performed. HPLC chromatograms, recorded after 5 h of incubation, showed the presence of only one radioactive species, with the retention time characteristic for each studied radioconjugate. Exemplary HPLC radiochromatograms for [^68^Ga]Ga-NODAGA-Bn-NH(CH_2_)_9_Tac and [^68^Ga]Ga-THP-NH(CH_2_)_9_Tac radioconjugates are presented in Figure 3.

### 2.3. Biodistribution Study

Among the studied radioconjugates, two radiocompounds, characterized by the highest LogD_7.4_ values (Table 1), have been selected for further biodistribution studies: [^68^Ga]Ga-NODAGA-Bn-NH(CH_2_)_9_Tac and [^68^Ga]Ga-THP-NH(CH_2_)_9_Tac.

**Figure 4:**
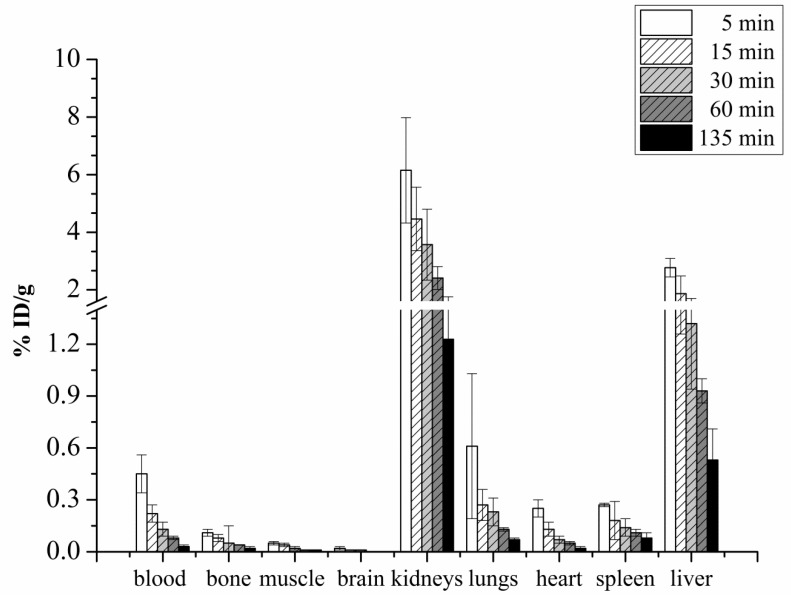
Biodistribution data of [^68^Ga]Ga-NODAGA-Bn-NH(CH_2_)_9_Tac in rats after different time p.i.

**Figure 5:**
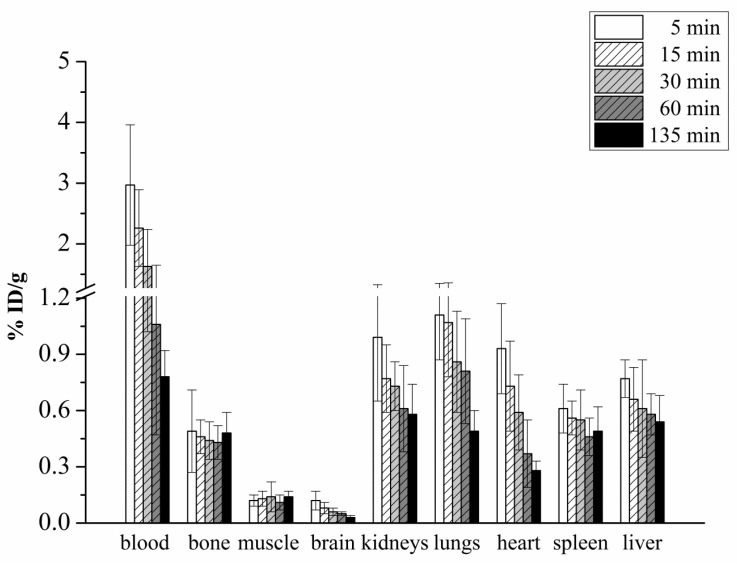
Biodistribution data of [^68^Ga]Ga-THP-NH(CH_2_)_9_Tac in rats after different time p.i.

**Figure 6:**
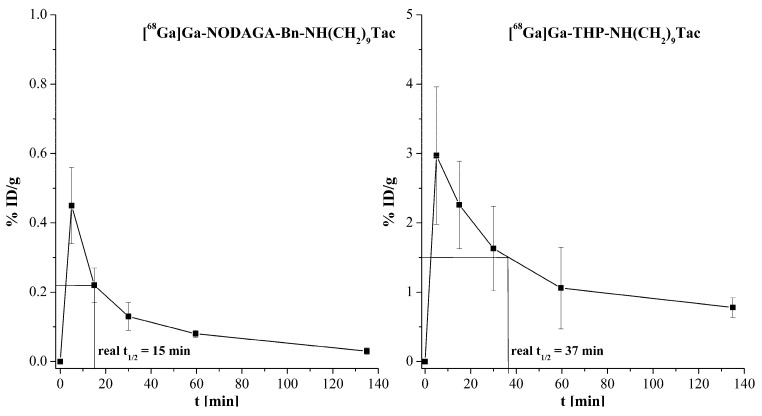
Results of [^68^Ga]Ga-NODAGA-Bn-NH(CH_2_)_9_Tac and [^68^Ga]Ga-THP-NH(CH_2_)_9_Tac pharmacokinetics in blood samples determined by ex vivo radioactivity measurements, with effective (real) half-lives of radioconjugates (15 min and 37 min, respectively) determined from the charts. To each plot an initial point of the coordinate system was added representing an absence of radioconjugate at 0 min time point.

Biodistribution data of [^68^Ga]Ga-NODAGA-Bn-NH(CH_2_)_9_Tac and [^68^Ga]Ga-THP-NH(CH_2_)_9_Tac are presented in Table 2 and Table 3 and in Figure 4 and Figure 5, respectively. Based on the obtained results, the effective half-life values of both radioconjugates have been graphically determined from the charts (Figure 6). All 50 rats used in the study have successfully passed through radiopharmaceutical application procedures without any noticeable side effects.

Based on the biodistribution profiles of radioconjugates [^68^Ga]Ga-NODAGA-Bn-NH(CH_2_)_9_Tac and [^68^Ga]Ga-THP-NH(CH_2_)_9_Tac, radiopreparation [^68^Ga]Ga-THP-NH(CH_2_)_9_Tac turned out to be a better radiopharmaceutical candidate. High uptake of this radioconjugate was observed in the blood, bone, kidneys, heart, spleen and liver (Table 3, Figure 5). However, the kidneys, spleen and liver are organs normally involved in the excretion of various substances from the organism and high uptake in these organs is practically always observed. A noticeably high uptake of radiopreparation in the blood and heart is related to its sustained circulation in the body, while visible uptake in bone is associated with the nature of the chelator (it is likely that chelator THP contains hydroxypyridinone groups that have an affinity for bone). Particularly noteworthy is the high uptake in the lungs, which allows us to draw conclusions on the specificity of the tested radioconjugate for this organ. For this reason, the study of this compound’s biological activity against cholinesterases was also performed.

### 2.4. ChE Inhibition Assay

Initially, the inhibition propensity of the radioconjugates on EeAChE (EC 3.1.1.7, acetylcholinesterase from Electrophorus electricus—Merck, Darmstadt, Germany) was qualitatively tested (using their reference compound—Tacrine—Tetrahydroaminacrine hydrochloride hydrate, Merck, Darmstadt, Germany) by the modified Ellman’s method. The tests confirmed that the studied radioconjugates retained the properties of acetylcholinesterase inhibition. This means that the structural modification of the tacrine derivative (chelator attachment and radionuclide labeling) does not affect the inhibitory properties of the tacrine derivatives in the tested range of concentration. Then, for the selected [^68^Ga]Ga-THP-NH(CH_2_)_9_Tac radioconjugate was determined (using Ga-THP-NH(CH_2_)_9_Tac reference compound in the study) its IC_50_ (50% inhibitory concentration) against EeAChE (electric eel acetylcholinesterase) and EqBuChE (EC 3.1.1.8, butyrylcholinesterase from equine serum—Merck, Darmstadt, Germany) (Table 4). Compared to the reference compound (tacrine), the new radioconjugate shows a much stronger activity towards both EeAChE and EqBuChE, and simultaneously lower activity against EqBuChE than EeAChE. Therefore, it can be considered as a new, attractive diagnostic agent, also in the context of the imaging of pathological conditions of the lungs due to the presence of cholinesterases in the lung glial tissue.

### 2.5. Molecular Modeling

**Figure 7:**
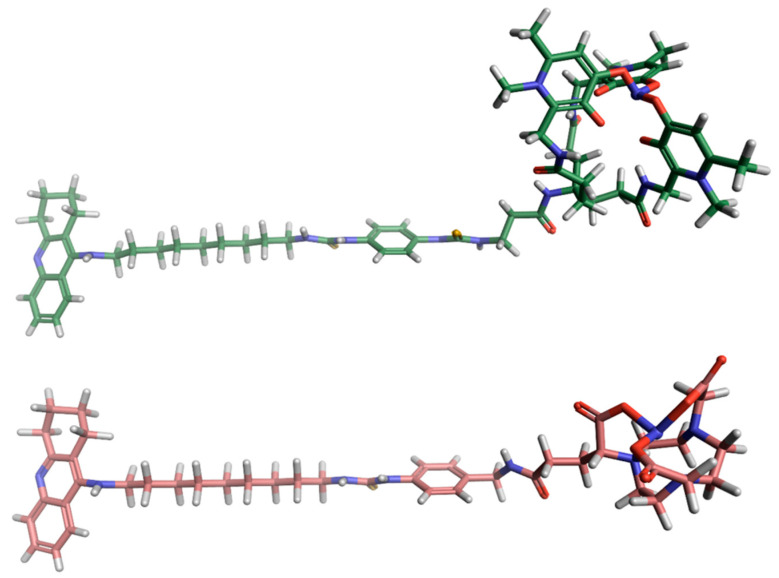
Three-dimensional structures of Ga-THP-NH(CH_2_)_9_Tac (**top**) and Ga-NODAGA-Bn-NH(CH_2_)_9_Tac (**bottom**) compounds, calculated at the DFT level of theory. Note, that tacrine and the nonamethylene chain are identical for both compounds, while the linkers, i.e., thiourea-phenyl-thiourea in Ga-THP-NH(CH_2_)_9_Tac and thiourea-phenyl in Ga-NODAGA-Bn-NH(CH_2_)_9_Tac, as well as chelators, i.e., THP in Ga-THP-NH(CH_2_)_9_Tac and NODAGA in Ga-NODAGA-Bn-NH(CH_2_)_9_Tac, are different.

In order to better document the validity of indicating [^68^Ga]Ga-THP-NH(CH_2_)_9_Tac radiopreparation as an effective potential radiopharmaceutical, studies of ligand-based in silico docking experiments of Ga-THP-NH(CH_2_)_9_Tac and Ga-NODAGA-Bn-NH(CH_2_)_9_Tac to the crystallographic structure of human acetylcholinesterase (hAChE) have been also performed. Three-dimensional structures of Ga-THP-NH(CH_2_)_9_Tac and Ga-NODAGA-Bn-NH(CH_2_)_9_Tac are presented in Figure 7.

**Figure 8:**
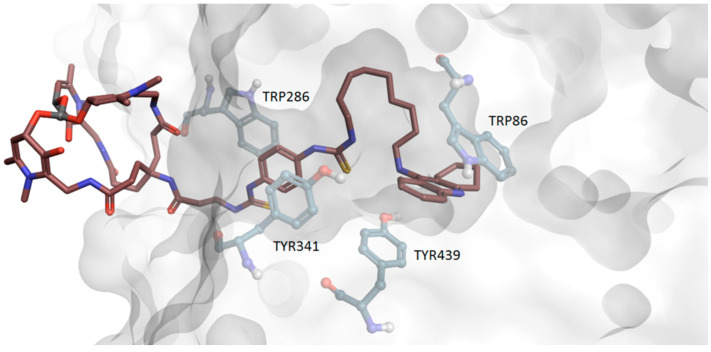

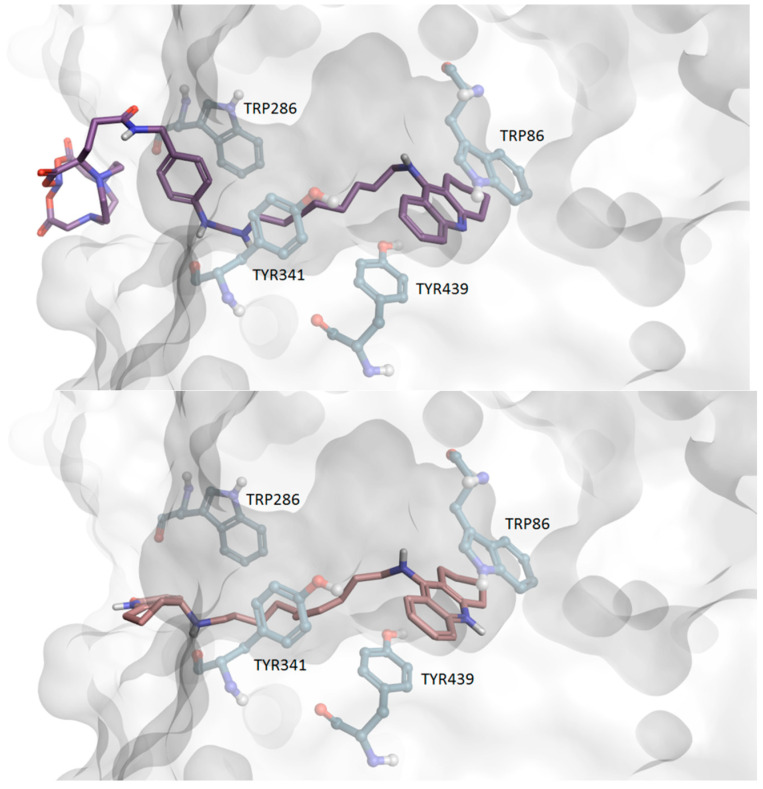
The side section of the hAChE’s binding pocket is docked with Ga-THP-NH(CH_2_)_9_Tac (**top**), Ga-NODAGA-Bn-NH(CH_2_)_9_Tac (**middle**), and the template ligand ((RS)-tacrine(10)-hupyridone) inhibitor (**bottom**), from the superimposed PDB id: 1ZGC structure. The key aromatic AChE’s residues involved in the pi-stacking interactions with ligands are in balls-and-sticks representation.

Results of the in silico docking experiments of Ga-THP-NH(CH_2_)_9_Tac and Ga-NODAGA-Bn-NH(CH_2_)_9_Tac compounds to the crystallographic structure of human acetylcholinesterase (hAChE) are presented in Figure 8.

## 3. Discussion

All radioconjugates were obtained with high yield and high radiochemical purity. Lipophilicity studies showed that two radioconjugates, namely [^68^Ga]Ga-NODAGA-Bn-NH(CH_2_)_9_Tac and [^68^Ga]Ga-THP-NH(CH_2_)_9_Tac were hydrophobic (the Log*D_7.4_* values of these radioconjugates were the highest in series of radioconjugates containing macrocyclic chelator and linear chelator, respectively), while the other were hydrophilic (Table 1). These two radioconjugates best fulfil the requirements for radiopharmaceuticals [28] and were selected for further examination.

Stability studies showed that all synthesised radioconjugates are almost completely stable in human serum (Figure 3). The HPLC chromatograms of both selected radioconjugates, recorded in a time interval corresponding to almost five radionuclide half-lives, showed single peaks with the retention time corresponding to the studied radioconjugate.

The qualitative studies of the inhibitory activity of the designed radioconjugates against AChE (performed as an initial study) still showed affinity for cholinesterases. The biodistribution performed for [^68^Ga]Ga-NODAGA-Bn-NH(CH_2_)_9_Tac and [^68^Ga]Ga-THP-NH(CH_2_)_9_Tac radioconjugates showed different biodistribution profiles of these two radiocompounds. The compound [^68^Ga]Ga-NODAGA-Bn-NH(CH_2_)_9_Tac accumulates mainly in excretory organs (kidneys and liver), and to a small extent, in the blood, lungs, heart and spleen (Table 2, Figure 4). Overall, the radiocompound is quickly cleared from the body—its effective (real) half-life is 15 min (Figure 6). The compound [^68^Ga]Ga-THP-NH(CH_2_)_9_Tac, apart from a large uptake in the excretory organs (kidneys, liver and spleen), accumulates to a high degree in the blood, as well as in significant and comparable degrees in the lungs and heart (Table 3, Figure 5). Its effective half-life (Figure 6) is more than twice as long (37 min). In the case of this radioconjugate, the high uptake in the lungs deserves special attention, which allows us to draw conclusions about the specificity of the studied radioconjugate for this organ. These observations confirm how effectively different studies complement each other from the point of view of using a given radioconjugate as a diagnostic agent. Therefore, the activity studies on isolated enzymes in terms of affinity, scintigraphy tests in laboratory, precise determination of the compound activity are advisable, however, only biodistribution studies show the actual place of radiocompound accumulation in the relevant organ. It can be concluded that the main goal is to obtain a preparation not only with the best biological activity, but also with sufficient affinity for a given organ. In our case, it was in the lungs that we demonstrated sufficiently high accumulation of radioconjugate [^68^Ga]Ga-THP-NH(CH_2_)_9_Tac, and thus the possibility of diagnosing pathological states of the lungs using this preparation.

Ligand-based in silico docking experiments to the crystallographic structure of human acetylcholinesterase (hAChE) were additionally performed to allow us to unravel why [^68^Ga]Ga-THP-NH(CH_2_)_9_Tac radioconjugate exhibits much better binding properties than [^68^Ga]Ga-NODAGA-Bn-NH(CH_2_)_9_Tac.

As presented in Figure 7, both compounds consist of tacrine, connected via a nonamethylene chain to different linker and radionuclide-complexing chelator residues.

The acetylocholinesterase’s binding pocket is ~20 Å deep and can be divided into peripheral, mid-gorge, and catalytic sites. According to results of the in silico docking experiment (Figure 8), the tacrine residues of both studied radiocompounds bind to the catalytic site of hAChE by aromatic interactions with TRP86 and TYR337 residues as well as via van der Waals interactions with residue TRP439.

The flexible, nonamethylene chain, present in both investigated tacrine derivatives, shall promotes favorable arrangement of the other parts of these ligands inside the binding pocket. Still, even subtle differences in scaffolds of both linker or/and chelator residues may be responsible for disparate affinities toward the receptor.

Thanks to the rigidity of the ~10 Å long, close-to-planar thiourea-phenyl-thiourea linker, which was confirmed by quantum chemical calculations, [^68^Ga]Ga-THP-NH(CH_2_)_9_Tac intercalates easily between aromatic residues of the peripheral and mid-gorge sites of the hAChE’s binding pocket, allowing the linker’s phenyl to align between the TYR341 and TRP286 residues, optimally for the parallel-displaced pi-stacking interactions as well as for strong hydrogen bonds of thiourea fragments with ASP74 and SER293 residues. The high flexibility of the nonamethylene chain not only places the joined [^68^Ga]Ga-THP-NH(CH_2_)_9_Tac’s residues in positions favorable for binding, but, more importantly, anchors this ligand to the pocket by coiling inside a niche formed in the hAChE’s catalytic site by ASN74, VAL73, TYR72, ASN87, PRO88, GLY124, SER125 and GLY126, simultaneously binding with those amino acids via van der Waals interactions and weak hydrogen bonds (e.g., with ASN87). The coiled structure of the nonamethylene chain is additionally stabilized by an intramolecular hydrogen bond between tacrine’s amine group and the adjacent thiourea’s sulfur. The ~7 Å distance from TRP286 to the entrance of the pocket is similar to the distance from the linker’s phenyl to the THP, which allows it to keep the bulky chelator at the entrance of the pocket. Although the THP chelator pokes out of the binding pocket, it sticks to the surface of the protein due to high electrostatic complementarity, hydrogen bonding between hAChE’s nitrogen electron donors from the peptide bonds and THP’s carbonyl oxygens as well as pi-stacking interactions between the THP’s heterocycles and the aromatic amino acids at the surface of the protein (e.g., HIS287).

Although the NODAGA chelator is less bulky than THP, it still cannot enter the AChE’s pocket and, similarly to THP, NODAGA sticks to the surface of the protein thanks to high electrostatic complementarity, hydrogen bonding between hAChE’s nitrogen electron donors from the peptide bonds or HIS287 and NODAGA’s carbonyl oxygens. Due to ~4 Å being a shorter (lacking one thiourea unit) linker, in order to accommodate tacrine in a favorable position in the active site (close to TRP86) and linker’s phenyl in the peripheral site (close to TRP286), the nonamethylene chain of [^68^Ga]Ga-NODAGA-Bn-NH(CH_2_)_9_Tac must be stretched along the pocket. Hence, the nonamethylene chain of [^68^Ga]Ga-NODAGA-Bn-NH(CH_2_)_9_Tac is not anchored as it is in case of [^68^Ga]Ga-THP-NH(CH_2_)_9_Tac. Additional stabilization of [^68^Ga]Ga-NODAGA-Bn-NH(CH_2_)_9_Tac inside hAChE’s pocket is provided by a hydrogen bond between the nitrogen from thiourea unit and the oxygen of the peptide bond of SER293 and van der Waals interactions between the nonamethylene chain and aromatic residues along hAChE’s pocket. Still, the unanchored [^68^Ga]Ga-NODAGA-Bn-NH(CH_2_)_9_Tac ligand will dissociate much more easily from the hAChE’s pocket than the anchored [^68^Ga]Ga-THP-NH(CH_2_)_9_Tac, which corresponds to much higher stability of the complex of the latter ligand with hAChE. The anchoring of the nonamethylene chain of [^68^Ga]Ga-THP-NH(CH_2_)_9_Tac ligand is promoted by the close-to-planar, long, and rigid thiourea-phenyl-thiourea linker, which allows the adjustment of the position of the linker’s phenyl between TYR341 and TRP286—the key aromatic residues for pi-stacking interactions situated in the mid-gorge and peripheral sites, respectively.

In summary, the disparity between affinities of the two in silico studied tacrine derivatives-based radioconjugates arises directly from the geometrical differences in the scaffolds of their linkers connecting the nonamethylene chain with the THP or NODAGA chelators, which impacts the structural adaptation of the nonemethylene chain inside the human acetylcholinesterase binding pocket.

## 4. Materials and Methods

Radionuclide-complexing chelators: (**a**) 2,2’-(7-(1-carboxy-4-((2,5-dioxopyrrolidin- 1-yl)oxy)-4-oxobutyl)-1,4,7-triazonane-1,4-diyl)diacetic acid (**NODAGA-NHS**), (**b**) 2,2′-(7-(1-carboxy-4-((4-isothiocyanatobenzyl)amino)-4-oxobutyl)-1,4,7-triazonane- 1,4-diyl)diacetic acid (**NODAGA-Bn-NCS**), (**c**) 2,2′,2”-(10-(1-carboxy -4-((4-isothiocyanatobenzyl)amino)-4-oxobutyl)-1,4,7,10-tetraazacyclododecane-1,4,7-triyl)triacetic acid (**DOTAGA-Bn-NCS**), (**d**) [(R)-2-Amino- 3-(4-isothiocyanatophenyl)propyl]-trans-(S,S)-cyclohexane-1,2-diamine-pentaacetic acid (**DTPA-CHX-Bn-NCS**), and (**e**) N^1^,N^7^-bis((3-hydroxy-1,6-dimethyl-4-oxo- 1,4-dihydropyridin-2-yl)methyl)-4-(3-(((3-hydroxy-1,6-dimethyl-4-oxo-1,4-dihydropyridin-2-yl)methyl)amino)-3-oxopropyl)-4-(3-(3-(4-isothiocyanatophenyl)thioureido)propanamido)heptanediamide (**THP-Bn-NCS**) were purchased from CheMatech (Dijon, France).

The solvents and other commercially available compounds were reagent grade, purchased from Merck (Darmstadt, Germany) and used without further purification. ^68^Ga (emitter β+, T_1/2_ = 67.7 min, E_βmax_ = 1.9 MeV, in the form of [^68^Ga]GaCl_3_ in 0.1 M HCl solutions) was eluted from the commercially available ^68^Ge/^68^Ga Eckert & Ziegler (Berlin, Germany) generator. Deionized water was prepared in a Hydrolab water purification system (Hydrolab, Straszyn, Poland). The 5,5’-dithiobisnitrobenzoic acid (DTNB), acetylthiocholine iodide (ATChI) (enzyme substrate) and tacrine were obtained from Sigma–Aldrich (Munich, Germany). Human serum was a gift from the Regional Centre for Blood Donation and Blood Treatment in Warsaw, Poland.

The high-performance liquid chromatography (HPLC) analyses, separations and purifications of synthesised compounds were carried out using a VWR-Hitachi LaChrom Elite HPLC system which consisted of a pump L2130, column thermostat L-2350, UV diode array detector (DAD) L-2455, the EZChrom Elite data system and core-shell reverse phase column Phenomenex (Warsaw, Poland). The radioactivity was monitored using a 3 × 3” NaI(Tl) scintillation detector Raytest Gabi Star (Straubenhardt, Germany). The gradient and HPLC conditions were as follows: solvent A, 0.1% (*v/v*) trifluoroacetic acid (TFA) in water; solvent B, 0.1% (*v/v*) TFA in acetonitrile. The HPLC analyses were performed using two systems: System 1: analytical Phenomenex Aeris^TM^ Peptide XB-C18 column, 3.6 µm, 100 Å, 150 × 4.6 mm, UV detection (220–400 nm) or γ-detection, gradient elution: 0–20 min: 5 to 70% solvent B, 20–25 min: 70% to 95% solvent B; 1.2 mL/min; System 2: semi-preparative Phenomenex Aeris^TM^ Peptide XB-C18 column, 5 μm, 100 Å, 250 × 10 mm, UV detection (220–400 nm), gradient elution: 0–15 min 5 to 70% solvent B, 15–20 min 70 to 95% solvent B; 3 mL/min.

Mass Spectrometry (MS) spectra were measured on the Bruker 3000 Esquire mass spectrometer equipped with ESI.

### 4.1. Syntheses of Conjugates

#### 4.1.1. Synthesis of Tacrine Scaffold

The NH_2_(CH_2_)_9_Tac scaffold, used for all investigated compounds, was synthesized according to the procedure described in reference [29]. The synthesis of tacrine derivative concerned obtainment of 9-chloro-1,2,3,4-tetrahydroacridine by direct heating the mixture of anthranilic acid and cyclohexanone in fresh POCl_3_. In the next step of the synthesis, a coupling of 9-chloro-1,2,3,4-tetrahydroacridine with two equivalents of the nonane-1,9-diamine and catalytic amounts of sodium iodide, in the presence of phenol at 180 °C, was performed. The crude product was then purified by flash chromatography or by HPLC to obtain the final compound. The purity of the final product was determined by TLC, IR, ^1^H NMR and MS as well. Finally, the purity of NH_2_(CH_2_)_9_Tac derivative was higher than 97%. The detailed description of the characterization of this tacrine analogue can be found in reference [18].

Analytical data for NH_2_(CH_2_)_9_Tac, IR (KBr) ν (cm^−1^): 1562.4, 2853.3, 2925.9, 3059.8, 3289.1; ^1^H NMR (CDCl_3_) (δ ppm.): 7.9–7.3 (4H, in aromatic ring of benzene), 3.9 (s, 1H, NH), 3.4 (t, 2H, NHCH_2_), 3.0 (s, 2H, in cyclohexane ring), 2.6 (d, 4H, CH_2_CH_2_NH_2_), 2.5 (s, 2H, in cyclohexane ring), 2.1 (s, 2H, NH_2_), 1.9 (m, 4H, in cyclohexane ring), 1.5–1.6 (m, 2H, NHCH_2_CH_2_), 1.4–1.1 (br, 10H, CH_2_CH_2_ in aliphatic hydrocarbon chain). 

MS (*m*/*z*): Calculated: 339.52, Found: 340.28 [M + H^+^]

#### 4.1.2. Synthesis of the Chelator-NH(CH2)_9_Tac Series

**Synthesis of NODAGA-NH(CH_2_)_9_Tac:** The 1 mg (2.95 μmol) of tacrine derivative NH_2_(CH_2_)_9_Tac dissolved in 25 μL of DMF was added to 3 mg (4.3 μmol) of NODAGA-NHS dissolved in 50 μL of DMF, next 1.5 μL (10.76 μmol) Et_3_N was added. The reaction mixture was stirred at room temperature (RT) overnight.

**Synthesis of NODAGA-Bn-NH(CH_2_)_9_Tac:** The 1.5 mg (4.42 μmol) of tacrine derivative NH_2_(CH_2_)_9_Tac dissolved in 30 μL of DMF was added to 2.3 mg (4.42 μmol) of NODAGA-Bn-NCS dissolved in 50 μL of DMF and immediately 2.46 μL (18 μmol) Et_3_N was added. Reaction mixture was stirred at RT overnight.

**Synthesis of DOTAGA-Bn-NH(CH_2_)_9_Tac:** The coupling reaction between DOTAGA-Bn-NCS and tacrine derivative NH_2_(CH_2_)_9_Tac in the presence of Et_3_N was obtained similarly as described above. The molar ratio of the reagents used in the coupling reactions was 1.1: 1: 4, respectively and the reaction mixture was stirred at RT overnight.

**Synthesis of DTPA-CHX-Bn-NH(CH_2_)_9_Tac:** To an Eppendorf tube containing 1.84 mg (5.42 μmol) of tacrine derivative NH_2_(CH_2_)_9_Tac in 30 μL of DMF, 3.22 mg (5.42 μmol) of DTPA-CHX-Bn-NCS in 50 μL of DMF and 3.02 μL (22 μmol) of Et_3_N were added. The reaction mixture was left overnight at RT.

**Synthesis of THP-Bn-NH(CH_2_)_9_Tac:** To an Eppendorf tube were added 4.25 mg (4.42 μmol) of THP-Bn-NCS dissolved in 20 μL of DMSO, 1.5 mg (4.42 μmol) of tacrine derivative NH_2_(CH_2_)_9_Tac in DMSO and 4 equiv. of Et_3_N. The reaction mixture was left overnight at RT.

Crude products (Scheme 1, Table 1) were purified on a semi-preparative HPLC column in system 2 and lyophilized, yield ≈ 70–90%.

Bearing in mind that preparations able to cross the blood–tissue barrier should be characterized with relatively high lipophilicity, only tacrine derivative, containing nine methylene groups in an aliphatic hydrocarbon (nonamethylene) chain have been used. Retention time values of chelator-NH(CH_2_)_9_Tac conjugates recorded in HPLC analyses are presented in Table 1.

Analytical data for NODAGA-NH(CH_2_)_9_Tac, (2,2’-(7-(1-carboxy-4-oxo-4-((9-((1,2,3,4-tetrahydroacridin-9-yl)amino)nonyl)amino)butyl)-1,4,7-triazonane-1,4-diyl)diacetic acid):

MS (FAB) *m/z*: Calculated for C_37_H_56_N_6_O_7_: 696.42; Found 697.42 [M + H^+^].

Analytical data for NODAGA-Bn-NH(CH_2_)_9_Tac, (2,2’-(7-(1-carboxy-4-oxo-4-((4-(3-(9-((1,2,3,4-tetrahydroacridin-9-yl)amino)nonyl)thioureido)benzyl)amino)butyl)-1,4,7-triazonane-1,4-diyl)diacetic acid):

MS (FAB) *m/z*: Calculated for C_45_H_64_N_8_O_7_S: 860.46; Found 861.39 [M + H^+^].

Analytical data for DOTAGA-Bn-NH(CH_2_)_9_Tac, (2,2’,2’’-(10-(1-carboxy-4-oxo-4-((4-(3-(9-((1,2,3,4-tetrahydroacridin-9-yl)amino)nonyl)thioureido)benzyl)amino)butyl)-1,4,7,10-tetraazacyclododecane-1,4,7-triyl)triacetic acid):

MS (FAB) *m/z*: Calculated for C_49_H_71_N_9_O_9_S: 961.51; Found 962.50 [M + H^+^].

Analytical data for DTPA-CHX-NH(CH_2_)_9_Tac, (2,2’-((2-((2-(bis(carboxymethyl)amino)-3-(4-(3-(9-((1,2,3,4-tetrahydroacridin-9-yl)amino)nonyl)thioureido)phenyl)propyl)(carboxymethyl)amino)cyclohexyl)azanediyl)diacetic acid): 

MS (FAB) *m/z*: Calculated for C_48_H_67_N_7_O_10_S: 933.47; Found 934.49 [M + H^+^].

Analytical data for THP-NH(CH_2_)_9_Tac, (N^1^,N^7^-bis((3-hydroxy-1,6-dimethyl-4-oxo-1,4-dihydropyridin-2-yl)methyl)-4-(3-(((3-hydroxy-1,6-dimethyl-4-oxo-1,4-dihydropyridin-2-yl)methyl)amino)-3-oxopropyl)-4-(3-(3-(4-(3-(9-((1,2,3,4-tetrahydroacridin-9-yl)amino)nonyl)thioureido)phenyl)thioureido)propanamido)heptanediamide):

MS (FAB) *m/z*: Calculated for C_67_H_89_N_13_O_10_S_2_: 1299.63; Found 1300.76 [M + H^+^]

#### 4.1.3. Preparation of [^68^Ga]Ga-chelator-NH(CH_2_)_9_Tac Radioconjugates

The [^68^Ga]Ga-chelator-NH(CH_2_)_9_Tac radioconjugates was synthesized according to the following procedure: 500–1000 µL of [^68^Ga]GaCl_3_ from the ^68^Ge/^68^Ga generator (100–220 MBq) was added into the vial containing about 50–100 µg of lyophilized chelator-NH(CH_2_)_9_Tac conjugate previously dissolved in 300 µL of a 0.2 M acetate buffer (pH = 5.0). The reaction mixture was heated for 15 min at 50 °C and the reaction progress was checked by HPLC method in system 1 with γ-detection. The radiochemical yield of the synthesized radioconjugates was higher than 95%. Retention time values of [^68^Ga]Ga-chelator-NH(CH_2_)_9_Tac radioconjugates recorded in HPLC analyses are presented in Table 1.

#### 4.1.4. Preparation of Reference Compounds Ga-chelator-NH(CH_2_)_9_Tac

In order to verify the identity of the [^68^Ga]Ga-chelator-NH(CH_2_)_9_Tac radioconjugates synthesised in n.c.a. scale, for all radioconjugates the non-radioactive reference compounds Ga-chelator-NH(CH_2_)_9_Tac were prepared in milligram scale (using GaCl_3_ solution and according to the procedure described above), isolated by HPLC method (system 1, UV detection) and characterised by MS analysis.

Analytical data for Ga-NODAGA-NH(CH_2_)_9_Tac (reference compound):

MS (FAB) *m/z*: Calculated for C_37_H_53_N_6_O_7_Ga: 762.32; Found 763.29 [M + H^+^]

Analytical data for Ga-NODAGA-Bn-NH(CH_2_)_9_Tac (reference compound):

MS (FAB) *m/z*: Calculated for C_45_H_61_N_8_O_7_SGa: 926.36; Found 927.30 [M + H^+^]

Analytical data for Ga-DOTAGA-Bn-NH(CH_2_)_9_Tac (reference compound):

MS (FAB) *m/z*: Calculated for C_49_H_69_N_9_O_9_SGa: 1028.42; Found 1028.41 [M + H^+^]

Analytical data for Ga-DTPA-CHX-NH(CH_2_)_9_Tac (reference compound):

MS (FAB) *m/z*: Calculated for C_48_H_64_N_7_O_10_SGa: 999.37; Found 1000.40 [M + H^+^]

Analytical data for Ga-THP-NH(CH_2_)_9_Tac (reference compound):

MS (FAB) *m/z*: Calculated for C_67_H_86_N_13_O_10_S_2_Ga: 1365.53; Found 1366.56 [M + H^+^]

### 4.2. Physicochemical Properties Studies of Novel Radioconjugates

All obtained radioconjugates were isolated by HPLC method (system 1, γ-detection) and then use in further experiments such as lipophilicity and stability studies. In case of in vivo studies Solid Phase Extraction method was performed using Sep-Pak^®^ classic short C18 cartridge, (WATERS, Milford, MA, USA), previously validated by HPLC (system 1). The reaction mixture was passed through the C18 cartridge previously conditioned with 3 mL of 95% ethanol followed by 10 mL deionized water. After that, the C18 cartridge was washed by 2–3 mL of deionized water and then the purified radioconjugate was eluted with 2mL 95% ethanol. The final solution was evaporated under N_2_ stream and dissolved again in the 0.05 M PBS buffer or 0.9% saline for later use.

#### 4.2.1. Lipophilicity Studies

Lipophilicity (expressed as Log*D*_7.4_) of all radioconjugates, which is an important factor affecting the distribution of drug molecules in the organism, was characterized by their distribution coefficients, *D*, in the system *n*-octanol/PBS buffer (pH = 7.4). The activity of each layer (which shows concentration of the radionuclide containing species in the layer) was determined by measuring γ-radiation, with a well-type NaI(Tl) detector (ISOMED 2100, NUVIA Instruments, Dresden, Germany). Distribution coefficient *D* was calculated as the ratio of radioactivity of organic to radioactivity of aqueous phase (as an average value from at least three independent measurements). It should be noted that immediately after each distribution experiment the aqueous phase was analyzed by HPLC in system 1 with γ-detection to check whether the studied radioconjugate has not decomposed during the experiment.

#### 4.2.2. Radioconjugates Stability Studies in Human Serum

Stability studies of all [^68^Ga]Ga-ligand-NH(CH_2_)_9_Tac radioconjugates were carried out as follows: 0.2 mL of solution of the radioconjugate (isolated from the reaction mixture using HPLC system 1 with γ-detection or Sep-Pak) in the 0.05 M PBS buffer (pH = 7.4), was added to 0.9 mL of human serum and incubated at 37 °C. At specified time intervals (up to few half-lives of the Ga-68) the content of the solution was tested by HPLC method. For that purpose, a small sample (0.2 mL) of the mixture was withdrawn, mixed with ethanol (0.5 mL) and vigorously shaken to precipitate proteins. Then, the sample was centrifuged (14,000 rpm, 5 min) and the supernatant was separated. To check if the radioconjugate did not convert into other water-soluble radioactive species during the experiment, aliquots of the supernatants were analyzed by HPLC for the content of the studied radioconjugate.

### 4.3. Biodistribution Studies of [^68^Ga]Ga-NODAGA-Bn-NH(CH_2_)_9_Tac and [^68^Ga]Ga-THP-NH(CH_2_)_9_Tac (Ex Vivo)

Biodistribution studies of the most promising [^68^Ga]Ga-NODAGA-Bn-NH(CH_2_)_9_Tac and [^68^Ga]Ga-THP-NH(CH_2_)_9_Tac radioconjugates were performed on an animal model of Wistar rats Crl:Cmd:(WI)WU). A total of 50 male rats in the age of 7–8 weeks were obtained from the animal facility at Mossakowski Medical Research Centre, Polish Academy of Sciences (Warsaw, Poland). All the experimental procedures were performed according to the national legislation and were approved by the First Local Ethical Committee of the Warsaw University Biology Department (Permission No. 510/2018 with further annex No. 943/2019). Rats were kept under constant conditions of 12 h-12 h light cycle, humidity at level of 55 ± 10%, and temperature of about 22 ± 2 °C in individually ventilated cages with free access to drinking water and standard laboratory diet. Any procedures were performed after a minimum 5 days of acclimation after travel and all efforts were made to minimise animals suffering.

Biodistribution study procedure was performed as static pharmacokinetics analysis for both radioconjugates by similarly performed intravenous applications of 4–15 MBq [^68^Ga]Ga-NODAGA-Bn-NH(CH_2_)_9_Tac or [^68^Ga]Ga-THP-NH(CH_2_)_9_Tac under isoflurane in oxygen anesthesia. Then in 5 specific time points (5, 15, 30, 60, 135 min) rats were sacrificed by decapitation after prior sedation by isoflurane. From each animal samples of blood (3 mL), bone, muscle, whole brain, both kidneys, lungs, heart, spleen and whole liver were removed. Next, each of the analyzed organs was weighted in order to calculate the radioactivity accumulated in 1 g of a given tissue. All samples were analyzed on WIZARD^2^ 2480 Automatic Gamma Counter (PerkinElmer, Inc., Waltham, MA, USA) in 60 s triple measurements with decay correlation protocol. Collected radioactivity data were converted into the tissue percent of initial dose applied into the rat divided by tissue mass in grams (%ID/g) and their averages were illustrated in the function of time points.

### 4.4. Molecular Modelling

The molecule builder–editor, implemented in the *Avogadro 1.2.0* software, was used for creation of three-dimensional structures of the investigated compounds [30]. Density functional theory method at B3LYP/6–311++G (d,p) level, implemented in the *Gaussian 09* package, was employed to gain insights into the molecular structure of the studied compounds (e.g., the lowest energy conformation and the molecular electrostatic potential) as well as to assess the planarity and rigidity of the linkers as well as flexibility of the nonamethylene chain in the studied compounds [31]. The molecular docking as well as analysis and visualization of results were performed using *Flare 5.0.0.* platform, on the basis of experimentally resolved PDB id: 7E3I and 1ZGC crystal AChE’s structures [32,33]. The first structure consists of human acetylcholinesterase (hAChE) in complex with tacrine, while the second one of *Torpedo californica* acetylcholinesterase in complex with tacrine derivative connected to a heterocycle by decamethylene chain. The preparation of the AChE model for docking, including cleaning the protein structures, adding hydrogens, protonation of histidines, removing ligands and water molecules etc., was performed using the protein-preparation tools implemented in *Flare* platform [34]. After superpositioning of the clean 1E3I and 1ZGC AChE’s structures, the ligand-based docking of the studied compounds to the protein structure of 1E3I was performed, using the tacrine-based ligand of the 1ZGC as the ligand template. The docking calculations were performed using the *Extra Precision Docking Algorithm* implemented in *Flare*. The results of the best ligand positions inside the AChE’s pocket with the lowest docking score obtained from *Flare* calculations were chosen for further analysis of the protein-ligand interactions.

### 4.5. Biological Activity Studies of [^68^Ga]Ga-THP-NH(CH_2_)_9_Tac Radioconjugate

The verification of acetylcholinesterase affinity of the synthesised Tac-based radiopharmaceuticals (using their reference compounds) was carried out using the modified Ellman’s method, with Tacrine as the reference compound. Two series of studies were performed here: initial qualitative studies for all five synthesized radioconjugates in a concentration range of 0.1 to 100.0 nM (the inhibitory affinity of each compound was qualified as a positive result when absorbance was higher than blank sample (without any compound)), and then quantitative research for the selected [^68^Ga]Ga-THP-NH(CH_2_)_9_Tac radioconjugate for nine test compound concentrations in the range 10–375 nM. All solutions were prepared in a phosphate buffer (PBS) at pH 8.0. In each experiment to the amount of 14 µL of a diluted compound solution 40 µL of acetylthiocholine iodide (Sigma–Aldrich) was added as a substrate. After adding 76 µL of Ellman’s reagent (5,5′-dithiobis-(2-nitrobenzoic acid), DTNB, Sigma–Aldrich), the reaction was initiated by addition of 10 µL of acetylcholinesterase (2 U/mL, AChE from the electric eel, Sigma–Aldrich, Munich, Germany) or butyrylcholineasterase (4 U/mL, BChE from equine serum, Sigma–Aldrich, Munich, Germany). The reference and tested compounds were incubated at various concentrations for 10 min at room temperature in 96-well plates. The production of the yellow anion was measured at a wavelength of 412 nm. All investigations were conducted in triplicates.

## 5. Conclusions

Viral pneumonia caused by highly infectious SARS-CoV-2 poses a higher risk to elderly people and those who have underlying health conditions, including Alzheimer’s disease. In this work, we presented newly designed tacrine derivative-based radioconjugates with physicochemical and biological properties that are crucial for the potential application as diagnostic radiopharmaceuticals targeting acetylcholinesterase. A set of five tacrine derivatives was synthesized, labelled with gallium-68 and fully characterized in the context their physicochemical properties. Based on these results, the final two most promising radioconjugates, [^68^Ga]Ga-NODAGA-Bn-NH(CH_2_)_9_Tac and [^68^Ga]Ga-THP-NH(CH_2_)_9_Tac, were selected for biodistribution studies. They both fulfill crucial criteria as potential diagnostic radiopharmaceuticals, which are strictly required from the clinical application point of view. Additionally, the biodistribution studies were performed using homemade conjugate kits reaching radiochemical yields of more than 95% in every case. Both selected compounds were proven by in vitro enzymatic assays to be effective inhibitors of cholinesterases, but, according to the in vivo biodistribution studies, only [^68^Ga]Ga-THP-NH(CH_2_)_9_Tac exhibited significant affinity toward the lungs. On the basis of molecular modelling combined with in vitro enzymatic studies, we unraveled which structural properties of the developed tacrine derivatives are crucial for high affinity toward acetylcholinesterase biomarkers of lung inflammation. The [^68^Ga]Ga-THP-NH(CH_2_)_9_Tac radiocompound, thanks to its increased accuracy and improved sensitivity in PET imaging of lung tissue with high levels of acetylcholinesterase, may become a novel potential radiopharmaceutical for the determination of lung perfusion, including inflammation after COVID-19. 

## Figures and Tables

**Table 1:** t_R_ values of conjugates (system 2, UV detection), radioconjugates (system 1, gamma detection) and reference compounds (system 1, UV detection), and logD_7.4_ values determined for radioconjugates.

Compound	Retention Time, t_R_ [Min]	Log D_7.4_
NODAGA-NH(CH_2_)_9_Tac	12.92	-----
[^68^Ga]Ga-NODAGA-NH(CH_2_)_9_Tac	12.33	−0.26 ± 0.01
Ga-NODAGA-NH(CH_2_)_9_Tac	11.65	-----
NODAGA-Bn-NH(CH_2_)_9_Tac	14.32	-----
[^68^Ga]Ga-NODAGA-Bn-NH(CH_2_)_9_Tac	13.89	−0.03 ± 0.02
Ga-NODAGA-Bn-NH(CH_2_)_9_Tac	13.47	-----
DOTAGA-Bn-NH(CH_2_)_9_Tac	13.54	-----
[^68^Ga]Ga-DOTAGA-Bn-NH(CH_2_)_9_Tac	13.54	−1.36 ± 0.02
Ga-DOTAGA-Bn-NH(CH_2_)_9_Tac	13.24	-----
DTPA-CHX-NH(CH_2_)_9_Tac	14.47	-----
[^68^Ga]Ga-DTPA-CHX-NH(CH_2_)_9_Tac	14.50	−0.83 ± 0.02
Ga-DTPA-CHX-NH(CH_2_)_9_Tac	13.85	-----
THP-NH(CH_2_)_9_Tac	14.09	-----
[^68^Ga]Ga-THP-NH(CH_2_)_9_Tac	14.11	0.73 ± 0.07
Ga-THP-NH(CH_2_)_9_Tac	13.61	-----

**Table 2:** [^68^Ga]Ga-NODAGA-Bn-NH(CH_2_)_9_Tac uptake in selected rat organs determined after different time periods by ex vivo radioactivity measurements.

	Time Period	[^68^Ga]Ga-NODAGA-Bn-NH(CH_2_)_9_Tac (Mean ± SD) [%ID/g]				
Organ		5 Min	15 Min	30 Min	60 Min	135 Min
blood		0.45 ± 0.11	0.22 ± 0.05	0.13 ± 0.04	0.08 ± 0.01	0.03 ± 0.01
bone		0.11± 0.02	0.08 ± 0.02	0.05 ± 0.01	0.04 ± 0.01	0.02 ± 0.01
muscle		0.05 ± 0.01	0.04 ± 0.01	0.02 ± 0.01	0.01 ± 0.00	0.01 ± 0.00
brain		0.02 ± 0.01	0.01 ± 0.00	0.01 ± 0.00	0.00 ± 0.00	0.00 ± 0.00
kidneys		6.15 ± 1.83	4.46 ± 1.10	3.57 ± 1.23	2.41 ± 0.40	1.23 ± 0.53
lungs		0.61 ± 0.42	0.27 ± 0.09	0.23 ± 0.08	0.13 ± 0.01	0.07 ± 0.01
heart		0.25 ± 0.05	0.13 ± 0.04	0.07 ± 0.02	0.05 ± 0.01	0.02 ± 0.01
spleen		0.27 ± 0.01	0.18 ± 0.11	0.14 ± 0.05	0.11 ± 0.02	0.08 ± 0.04
liver		2.77 ± 0.32	1.87 ± 0.61	1.32 ± 0.38	0.93 ± 0.07	0.53 ± 0.18

**Table 3:** [^68^Ga]Ga-THP-NH(CH_2_)_9_Tac uptake in selected rat organs determined after different time p.i. by ex vivo radioactivity measurements.

	Time Period	[^68^Ga]Ga-THP-NH(CH_2_)_9_Tac (Mean ± SD) [%ID/g]				
Organ		5 Min	15 Min	30 Min	60 Min	135 Min
blood		2.97 ± 0.99	2.26 ± 0.63	1.63 ± 0.61	1.06 ± 0.59	0.78 ± 0.14
bone		0.49 ± 0.22	0.46 ± 0.09	0.44 ± 0.1	0.43 ± 0.09	0.48 ± 0.11
muscle		0.12 ± 0.03	0.13 ± 0.04	0.14 ± 0.08	0.11 ± 0.04	0.14 ± 0.03
brain		0.12 ± 0.05	0.08 ± 0.03	0.06 ± 0.02	0.05 ± 0.03	0.03 ± 0.01
kidneys		0.99 ± 0.34	0.77 ± 0.18	0.73 ± 0.13	0.61 ± 0.23	0.58 ± 0.16
lungs		1.11 ± 0.24	1.07 ± 0.29	0.86 ± 0.27	0.81 ± 0.28	0.49 ± 0.11
heart		0.93 ± 0.24	0.73 ± 0.24	0.59 ± 0.20	0.37 ± 0.18	0.28 ± 0.05
spleen		0.61 ± 0.17	0.56 ± 0.09	0.55 ± 0.26	0.46 ± 0.11	0.49 ± 0.13
liver		0.77 ± 0.11	0.66 ± 0.17	0.61 ± 0.16	0.58 ± 0.11	0.54 ± 0.14

**Table 4:** Cholinesterase inhibitory activities of [^68^Ga]Ga-THP-NH(CH_2_)_9_Tac radiocompound and tacrine as a reference compound.

Compound	IC_50_ Values ± SD [nM]	
	EeAChE	EqBuChE
[^68^Ga]Ga-THP-NH(CH_2_)_9_Tac	0.186 ± 0.012	0.878 ± 0.103
Tacrine	89.429 ± 5.103	48.036 ± 4.674

## Data Availability

Not applicable.
